# Media News Engagement and Parent–Child Well-being during Regional Conflict: A Daily Diary Study

**DOI:** 10.1007/s10802-026-01470-x

**Published:** 2026-06-17

**Authors:** Yael Dann, Noa Gueron-Sela

**Affiliations:** The Department of Psychology, Ben-Gurion University of the Negev, Negev P.O.B. 653, 8410501 Beer-Sheva, Israel

**Keywords:** Parent–child relationship, Media use, War exposure, Diary study, Child well being

## Abstract

In the current media-rich environment, exposure to war and terror often occurs indirectly through various media platforms. While extensive research shows that news media engagement adversely impacts adults’ emotional states, the effects on young children remain underexplored. Utilizing a 7-day daily diary design, this study examined the effects of war-related media exposure on parent and child well-being during active regional conflict. Participants were 193 Jewish-Israeli parents (child Mage = 4.53 years, 53.4% girls; parent Mage = 38 years, 51.3% fathers), who provided daily reports on both themselves and their children on war-related media exposure, direct exposure to war, negative affect and parent–child relational strain. Multilevel models revealed that children’s higher media exposure was associated with increased negative affect and more parent–child relational strain both at the between-participants and the within-participants levels. The associations between parental media engagement and child negative affect were mediated by parental negative affect and parent–child relational strain. Finally, children’s direct exposure and media exposure to war interacted to predict their well-being. These findings underscore the significant role of parent and child media exposure in shaping the emotional climate within families during times of war and related acts of violence.

**Supplementary Information:**

The online version contains supplementary material available at 10.1007/s10802-026-01470-x.

## Introduction

Residing in conflict-affected regions has a significant impact on the well-being of both adults and children (Garcia et al., 2021; Slone & Shoshani, 2022). Children exposed to war-related events often exhibit disruptions across social-emotional and cognitive domains, frequently presenting with post-traumatic stress reactions (Joshi & O’donnell, 2003). This exposure can occur both directly—through firsthand experiences such as proximity to violence—and indirectly, as individuals engage with news about these events through various media platforms, including television, apps, and social media. In today’s media-saturated environment, the media plays an increasingly pivotal role in disseminating news and portraying war-related incidents (Comer & Kendall, 2007). While most studies on news engagement have primarily focused on adults and their emotional responses, there is a growing acknowledgment of the effects of media exposure on young children, with increased exposure being associated with elevated fear and anxiety (Holman et al., 2020a, 2020b; Joshi & O’donnell, 2003; Pfefferbaum et al., 2018; Riddle et al., 2012; Wang et al., 2006; Wilson, 2008). However, significant gaps remain in understanding how both parent and child war-related media exposure is related to their emotional well-being. To address, this issue, this study aimed to explore the mechanisms through which parental and child news exposure impacts their affective states, and the interplay between direct and indirect (through media) exposure to war-related events on both children and parents.

### War-related Media Engagement and Psychological Functioning

During periods of armed conflict or terror events, individuals are inundated with distressing media content, including graphic images and videos of violent acts, disseminated continuously through various media platforms (e.g., Su et al., 2023). This exposure is often exacerbated by algorithm-driven social media platforms, amplifying its reach and impact (Makhortykh & Bastian, 2022). Regardless of their physical proximity to conflict zones, individuals are confronted with the stark realities of war and the potential psychological trauma it entails. For example, a study examining media consumption following the 2015 terror attack in Paris showed that engagement with both social media and traditional news outlets was related to heightened psychological distress and insomnia (Goodwin et al., 2018; Monfort & Afzali, 2017). Research investigating media exposure after the September 11, 2001, terror attacks and during the beginning of the Iraq War in 2003 highlighted that prolonged television viewing of war and terror-related content predicted enduring symptoms of post-traumatic stress disorder (PTSD), observable even three years later (Silver et al., 2013). In a study comparing direct and indirect exposure to the 2013 Boston Marathon bombings, individuals who reported six or more daily hours of bombing-related media exposure reported higher levels of acute stress compared to those directly impacted by the bombings (Holman et al., 2014). Research has also identified differences between voluntary vs. involuntary news engagement. For example, a study examining media exposure during the Russia-Ukraine conflict found that individuals with higher levels of involuntary exposure to war-related news exhibited more severe stress symptoms than those who sought information about it voluntarily (Castro et al., 2023). While most prior research has focused on adults, children are often exposed to such content through shared family media environments and have limited ability to regulate or filter their media consumption; thus, it is critical to examine how war-related media exposure is associated with their psychological functioning. Additionally, exposure to war-related media during ongoing conflicts is rarely a single event. Rather, individuals are repeatedly exposed to evolving news coverage and social media updates across days and weeks. However, most prior research has relied on cross-sectional or between-person approaches that capture overall levels of media exposure, potentially overlooking meaningful day-to-day fluctuations in engagement during prolonged crises; therefore, examining these fluctuations may provide important insight into how war-related media exposure is associated with emotional functioning (e.g., Holman et al., 2014; Silver et al., 2013).

### Children’s Exposure to War-related Media

During early childhood, when physiological, cognitive, and emotional systems develop rapidly, exposure to frightening or graphic content such as extreme acts of violence shapes children’s perception and experience of potentially traumatic events. Cantor and Wilson (1984) emphasize that children’s comprehension of media content evolves with age; children aged 3–5 often lack the cognitive skills to process frightening stimuli effectively, unlike their older counterparts (9–11 years), whose emotional responses can be modified through external mediation. At the same time, evidence suggests that children in early middle childhood (6–8 years) may also struggle to make sense of intense media coverage of terrorism and may continue to experience fear months later, often relying on media and peers as primary sources of information (Jørgensen et al., 2015). This developmental difference highlights heightened vulnerability in younger children, who are more likely to misinterpret the causes of distressing events, often attributing them to their own actions or beliefs (Lieberman & Knorr, 2007).

Studies investigating the effects of exposure to war through media have found significant impacts on children’s mental health (Pfefferbaum et al., 2018). For instance, a study focusing on television viewing habits of children and adults after the September 11 attacks found that young children under the age of 10 who watched more television on the day of the attacks were more likely to exhibit full or subsyndromal PTSD symptoms compared to their peers who watched less television (Otto et al., 2007). While much of the existing research on media of exposure to terror events has focused on older children (Pfefferbaum et al., 2019), it is crucial to consider the effects on younger age groups. Studies like Wang et al. (2006) indicate that preschool children exposed to terror-related media are at increased risk for externalizing problems, heightened emotional reactivity, sleep disturbances, aggressive behavior, and oppositional defiant issues. Research on trauma and violence exposure in this age group consistently demonstrates severe and detrimental effects, underscoring the urgent need to investigate this vulnerable population further (Lieberman & Knorr, 2007).

Despite the proliferation of news engagement through apps and social media, most studies on reactions to war media coverage focus on television viewing (e.g., Otto et al., 2007; Silver et al., 2013). Furthermore, research on young children’s news exposure tends to focus on the aftermath of single terrorist attacks rather than on children living in areas of sustained conflict. Because exposure to war-related media in family environments may vary across days, children’s indirect exposure to such content may also fluctuate. Given the limited research on children aged 3–5 years and their heightened vulnerability to the effects of media exposure, focusing on this age group allows us to deepen our understanding of how exposure to war-related news may significantly influence their emotional and psychological development, particularly given that such exposure may vary across days in the context of ongoing conflict.

### Children’s Indirect Exposure to War Through Parental Media Engagement

Previous research has highlighted the role of parents’ emotional states in the context of exposure to terror, as a significant factor that affects children’s well-being. Studies investigating post-traumatic stress reactions in children following events like the September 11 attacks have shown associations between parental reactions and children’s responses. For example, Fairbrother et al. (2003) found that children of parents exhibiting PTSD symptoms following the terror attack were four times more likely to experience similar symptoms. Studies on Israeli and Palestinian families exposed to continuous regional conflict also provide evidence for intergenerational effects of parents’ war-related trauma on children’s mental health (Halevi et al., 2017; Palosaari et al., 2013). However, despite acknowledging both the significant impact of parental exposure to news on their well-being (Holman et al., 2014; Monfort & Afzali, 2017) and the influence of parents’ emotional states on children’s well-being during war-related events, the indirect effects of parental news media engagement on their children have not yet been studied.

### Mechanisms Through Which Parental War-related Media Engagement Can Affect Children

Parental engagement with news media has the potential to influence children’s well-being through various pathways. Family systems theories offer a robust theoretical framework for examining these mechanisms. According to these theories, families function as interconnected systems in which the emotional and psychological states of one member can affect those of others (Minuchin, 1985; White & Klein, 2002). Specifically, the spillover hypothesis suggests that affect or behavior transfers directly from one individual or relationship to another within a family system. Transfer occurs in the same valence, such that negative affect in one member or subsystem is linked to negative affect in another. When parents engage with negative news-related media content, they may experience heightened levels of stress, fear, or anger (Holman et al., 2020a, 2020b; Pfefferbaum et al., 2021). Consequently, children may also experience increased distress and anxiety through direct transfer of negative affect, as studies on trauma and disasters consistently show strong links between child and parent mental states (Cobham et al., 2016; Lambert et al., 2014). Parental news media engagement can also have spillover effects through shaping the family environment and dynamics. Parental media use can reduce the availability and quality of parent–child interactions (Brito et al., 2017; Krapf-Bar et al., 2022) and foster preoccupation with distressing media content and negative emotions, potentially leading to negative parent–child interactions and negative child outcomes (Hopwood & Schutte, 2017). For example, elevated levels of perceived family conflict following a disaster have been linked to heightened symptoms of PTSD in youth (Bokszczanin, 2008), consistent with reports following the September 11 attacks (Gil-Rivas et al., 2007). Similarly, in a study involving primary school-aged children exposed to the 2010 Chilean earthquake, parent–child conflict, as perceived by children, was positively associated with child-reported post-traumatic stress symptoms (Garfin et al., 2014). Thus, parental news engagement may affect children through two distinct mechanisms: the affective state of the parent and changes in family dynamics that can manifest in strained parent–child relational processes. It’s crucial to distinguish between these mechanisms to clarify their unique impacts on children’s well-being.

### The Interplay Between Direct Exposure and Media Exposure to War-related Events

While many studies have underscored the physiological and health ramifications of both direct and media exposure to war-related events, few have delved into the interaction between these exposures. One exception is a study showing that young adults with a history of actual exposure to political and terrorism-related events experienced higher anxiety levels after watching terrorism-related television broadcasts compared to unexposed individuals (Slone et al., 2008).

Young children often display vague and somatic reactions to war-related and terror events. Their cognitive, social, and emotional capacities, which are still developing, make them particularly vulnerable to the detrimental effects of both direct and media exposure to violence (Joshi & O’donnell, 2003; Wang et al., 2006). However, the interplay between these two types of exposures remains largely unexplored.

### The Current Study

In October 2023, a large-scale terror attack occurred in Israel, marking the onset of prolonged regional conflict and a period characterized by intensified exposure to violent and distressing content across digital media platforms (Katsoty et al., 2024; Levany et al., 2023). During this time, graphic war-related materials depicting acts of violence were widely disseminated online, including images and videos circulated through social media and news outlets (Karniel & Lavie-Dinur, 2024; Weimann & Weimann-Saks, 2024). The widespread exposure of civilians to both direct and media coverage of war-related events offers a unique opportunity to investigate the effects of such exposure on individuals’ affective states.

Although literature has explored the impact of both direct and media exposure to terrorist events on adults and children (Betancourt et al., 2009; De Jong, 2006; Holman et al., 2014; Monfort & Afzali, 2017; Osokina et al., 2023; Otto et al., 2007), previous studies have primarily focused on between-person effects, neglecting to examine daily fluctuations in news engagement during ongoing exposure to war (i.e., within-person effects). Yet exposure to war-related media in ongoing conflict is inherently dynamic, varying across days depending on news cycles, ongoing security events, and individuals’ media engagement. Daily diary studies have provided important insights into how day-to-day variations in individuals’ experiences relate to fluctuations in emotional functioning and interpersonal processes (e.g., Boynton & O’Hara, 2018; Totenhagen et al., 2013). Examining these day-to-day fluctuations may therefore help clarify how variations in war-related media engagement relate to daily emotional responses and parent–child interactions, allowing a more precise understanding of the short-term links between war-related media exposure and families’ emotional and relational functioning during ongoing conflict. To capture these dynamic processes, the present study employed a daily diary design. Diary methods allow researchers to examine both between-person differences and within-person fluctuations across time, providing ecologically valid insights into individuals’ everyday experiences (Boynton & O’Hara, 2018).

Moreover, despite children’s heightened vulnerability to war exposure (Joshi & O’donnell, 2003) and the relationship between parental and children’s mental health (Cobham et al., 2016; Lambert et al., 2014), there is a notable absence of research examining the potential influence of parental news media engagement on children.

To address these questions, the present study examined both overall exposure and day-to-day fluctuations in war-related media engagement. Accordingly, the first aim of the present study was to examine the effects of parental exposure to war, including both direct exposure to war (i.e., the overall level of exposure to war-related incidents experienced during the ongoing conflict) and engagement with war-related news media, on parental and child well-being. Specifically, whether parents’ direct and media exposure to war-related events are associated with higher parental negative affect and more parent–child relational strain (H1); whether the association between parental news media engagement and children’s negative affect is mediated by parental negative affect and parent–child relational strain (H2); and whether direct exposure to war moderates the association between parental news media engagement and negative affect among parents and children (H3).

The second aim was to examine the effects of children’s exposure to war-related media on their negative affect and interactions with parents. Specifically, whether children’s direct and media exposure to war-related events is associated with higher negative affect and more parent–child relational strain (H4), and whether direct exposure to war moderates the association between children’s war-related media exposure and their negative affect and parent–child relational strain (H5).

## Methods

### Study Design

This study employed a 7-day diary design to capture daily fluctuations in parental and child news exposure through media and their associations with daily negative affect and parent–child relational strain. The decision to conduct the study over 7 days was motivated by its common usage in daily diary studies (e.g., Curran et al., 2015; Totenhagen et al., 2013; Vandewalle et al., 2017; Young et al., 2013) and the aim to maximize participant retention while minimizing potential fatigue (Bolger et al., 2003).

### Participants

The study protocol was reviewed and approved by the IRB committee at Ben-Gurion University of the Negev, Israel. Data collection occurred in Israel, between January 14th and February 4th, 2024. This period was marked by significant developments in the ongoing violent conflict between Israel and other regional actors.

Participants were recruited through the ‘Panel4all’ platform. Panel4all is an Israeli online research platform with access to approximately 30,000 panelists engaged in various online studies for remuneration (https://www.panel4all.co.il/). Due to its capabilities of screening participants on prescreening questions and conducting longitudinal studies by re-approaching them from the same source, Panel4all provides a valuable alternative to other crowdsourcing platforms and social media recruitment.

Participants were eligible if they were parents of a child aged 3–5 years. Parents meeting this criterion were approached through Panel4all and invited to participate. Parents were instructed to select one specific child within the specified age range and to complete questionnaires about themselves and the target child for seven consecutive days. Parents were instructed to consistently reference the same target child across all the days, confirming this by providing the child’s initials and age each day. To ensure that the same child was not reported on by two different parents, we cross-checked participants’ reported residential region, child age, and the initials of the target child across entries.

For the analysis, data from participants who completed questionnaires on at least five days within a one-week period were included. Initially, 313 subjects filled the baseline questionnaire and the first day questionnaire, 84 of whom were excluded due to lack of complete data for at least 5 days, resulting in 229 valid diary participants. Further exclusions were made: six subjects were omitted due to inconsistencies in completing questionnaires for the target child, as indicated by different child initials and age, eleven participants were excluded due to child health and developmental problems, and nineteen participants were excluded due to incorrect responses to attention-verification items on at least one occasion. Additionally, nine participants who provided responses on more than five days had one day of data excluded; either due to incorrect responses to attention-verification items (n = 8) or because they completed the questionnaire for a different target child on the same day (n = 1), so they still had at least five valid days. Ultimately, the final sample size consisted of 193 participants. Participants completed an average of 5.62 daily questionnaires (SD = 0.67; range = 5–7) out of a possible seven days; 94 participants completed five diaries, 78 completed six, and 21 completed all seven. Although participation was not restricted by sociodemographic criteria, only Jewish Israeli participants responded to the Panel4All recruitment invitation; accordingly, the present study focused on this population in Israel. Demographic characteristics of participants are presented in Table 1. Overall, the sample reflects variability across several key sociodemographic characteristics, including geographic region, education level, household income, and religiosity, and broadly mirrors the diversity of the Jewish Israeli population (Israel Central Bureau of Statistics, 2023).

**Table 1:** Demographic characteristics of participants

	*M*	*SD*	*Median*
Child gender (percent)			
girls	53.36%		
boys	46.64%		
Child age (years)	4.56	**0.8**	4.5
Parent gender (percent)			
Women	48.7%		
Men	51.3%		
Parental age (years)	38	5.44	38
Parental education (percent)			
Less than a school diploma	5.7%		
High-school Diploma	9.85%		
Post-secondary non-tertiary	20.2%		
Bachelor’s degree	42.5%		
Master’s degree	20.72%		
Doctoral degree (PhD)	1.03%		
Parent in active military service (percent)	9.3%		
Family monthly income (percent; relative to the national average monthly household income in Israel; 19,118 NIS)			
Much less than average	11.9%		
Less than average	16.6%		
Average	29.05%		
More than average	29.05%		
Much more than average	13.4%		
Place of residence			
Northern District	9.3%		
Haifa District	17.1%		
Center District	32.1%		
Tel-Aviv District	10.3%		
Jerusalem District	11.92%		
Southern District	8.8%		
Judea and Samaria Area	7.8%		
Other	2.6%		
Childcare setting (percent)			
Municipal preschool	85.0%		
Private preschool or nanny/home-based care	14.0%		
Other	1.0%		
Family status			
Married/Cohabiting	94.3%		
Single	2.1%		
Divorce/Seperated	3.6%		

### Procedure

Participants engaged in the study throughout the week, from Sunday to Saturday. On the first day, parents completed a baseline questionnaire and a daily questionnaire, while in subsequent days they responded only to the daily questionnaire. The baseline questionnaire encompassed demographic information, as well as direct exposure to war-related events. The daily questionnaire captured parent and child negative affect, strained parent–child interactions, and news media exposure on a particular day. Attention-verification items (e.g., "If you read this, please mark 4") were included on all days. Participants completed the daily diary measure starting at 7:00 PM, with reminders sent via text messages and emails. The questionnaire remained open until 7:00 AM the following morning.

### Measures

#### Objective Exposure to War-related Events

##### Overall Objective Exposure

Overall objective exposure to war-related events since the beginning of the Israel-Hamas war was assessed at baseline using the Exposure to Terror Questionnaire (Lavi, 2004), adapted to the specific context of the current regional conflict situation. The questionnaire consisted of 18 statements related to various traumatic incidents associated with terrorism and war, designed to capture a broader spectrum of experiences related to the current conflict. Parents were asked to indicate (i.e., “yes”/“no”) whether each statement corresponded to events experienced by themselves or their child (e.g., "Rocket alarms went off in our residential area"). The full questionnaire is available in the supplementary material. Objective exposure to war was calculated as the total number of incidents reported for the participant, ranging from 0 to 18.

##### Daily Objective Exposure

Parents were daily queried regarding war-related events that occurred on the same day. In contrast to the baseline assessment, the daily measure consisted of a single yes/no question asking whether the parent or child experienced any war-related event on that specific day. If participants responded ‘yes’, they were asked to specify the nature of the event, with examples including terrorist attack in my vicinity, rocket alarms in my area, loudly sounds of artillery, or being close to soldier killed in the fighting in Gaza, among others.

#### Daily War-related Media Exposure

##### Parent

Parents indicated their engagement with various media platforms including: Watching the news on TV/mobile phone/computer, reading news through news apps or websites on the mobile phone, listening to the radio, and reading or writing posts related to the war on social media platforms such as Facebook, Instagram, Twitter, TikTok, etc., as adapted from Beckers et al., 2021. Participants were instructed to rate their engagement level for each platform on a scale ranging from 1 to 6 (1 = no usage at all, 2 = less than 15 min, 3 = 15–30 min, 4 = 30–60 min, 5 = 1–2 h, 6 = more than two hours). Although news exposure items were phrased in general terms, it is important to note that during the study period in Israel media coverage was overwhelmingly dominated by the ongoing war (Karniel & Lavie-Dinur, 2024). Therefore, daily exposure to news media during this period largely reflected exposure to war-related content.

##### Child

Parents also reported the duration of time their child spent with them during these news engagement activities, using the same scale. This item referred to the child being present with the parent during news consumption and did not distinguish between different forms of exposure. Furthermore, participants reported on the presence of war-related background television during the last day, which refers to the duration the television was on for news purposes, regardless of viewership, on a scale ranging from 1 (not at all) to 4 (over two hours).

To calculate exposure to war-related media from all platforms and background television, scores were summed to obtain a total score for both parents and children separately.

#### Negative Affect and Parent–Child Relational Strain

##### Child Daily Negative Affect

Parents reported the frequency of five behaviors (sadness, irritability, tearfulness, tantrums, and anxiety) experienced by their child daily on a 5-point scale ranging from 1 (very little or not at all) to 5 (very much), as adapted from Bufferd et al. (2017). These behaviors were based on items derived from two established and reliable measures: the Early Childhood Inventory (Gadow & Sprafkin, 2000) and the Preschool Age Psychiatric Assessment (Egger et al., 1999). Daily negative affect was calculated as a composite of these five items (α = 0.87).

##### Parent Daily Negative Affect

Parental daily mood was assessed using a shortened version of the Negative Affect scale derived from the Positive and Negative Affect Schedule (Jones et al., 2007; Watson et al., 1988). Parents rated six negative emotions daily on a scale ranging from 1 (very slightly or not at all) to 5 (extremely): fear, stress, sadness, anger, worry, and unease. Parental daily negative affect was calculated as a composite score of these six items (α = 0.93).

##### Daily Parent–Child Relational Strain

Parents indicated daily whether (a) they found it hard to assert themselves concerning their child, (b) they found taking care of their child exhausting, and (c) they had disagreements with their child (Schmidt et al., 2021). These three items were summed to calculate a daily parent–child relational strain score (α = 0.84).

##### Covariates

Child and parent gender were included as covariates based on their associations with media consumption, news consumption, and mood (Lemish & Alony, 2014; Soroka et al., 2016; Toff & Palmer, 2019). Additional covariates were selected using a stepwise regression model for each outcome variable separately (Collignon & Monnez, 2016).

Based on the findings of the stepwise regression (see supplementary material), for the prediction model of child negative affect, ‘parent in military service’ and ‘child age’ were chosen as covariates. In the models examining parent–child relational strain and parent negative affect, ‘childcare’ (indicating whether the child attended childcare on the same day) was selected as covariate. Thus, child age, parent gender, child gender, and childcare attendance were included as covariates in all models.

### Analytic Approach

Prior to the main analysis, we inspected the distributions of the key study variables. Some skewness was observed, which is common in daily diary measures. Because multilevel models are generally robust to moderate deviations from normality in predictor variables (Gelman & Hill, 2007), no transformations were applied. Due to the data’s multilevel structure (days nested within families), we used a series of 2-level (a within-family level and a between-family level) multilevel models (MLMs) which were fitted using restricted maximum likelihood (REML), robust to missing data. All models were estimated by using R version 4.3.3 software (R Core Team, 2024).

Because the models distinguish between within-family (day-to-day fluctuations) and between-family differences in media exposure, the within-family effects capture deviations from each parent’s own average level of media engagement. These estimates are therefore less likely to be driven by stable individual differences in parents’ reporting styles (Bauer et al., 2006). In addition, the daily diary design reduces retrospective reporting bias by assessing experiences close in time to their occurrence (Bolger et al., 2003).

#### Between and Within Effects of War-related Media Exposure

To test our hypotheses regarding the direct effects of war-related media exposure (i.e., Hypothesis 1 and 4), we ran five 2-level growth MLMs: three models predicting child negative affect, parent negative affect, and parent–child relational strain from parental media news engagement and two models predicting child negative affect and parent–child relational strain from child news exposure. Models included both the between-family and within-family effects of parent and child media exposure, the between-family effect of overall exposure to war events, and between-family covariates (i.e., child gender, parent gender, child age, and parent in military service). Additionally, we controlled for within-family variables including childcare attendance and exposure to war events on a given day.

Following Hofmann and Gavin’s (1998) centering technique, day-level variables were centered on the mean of the individual to reduce variance between individuals. Person-level factors were based on grand-mean centering. Specifically, the categorical variables (child gender, parent gender, war events (yes/no), childcare (yes/no), and parent in military service (yes/no)) were centered around zero (0.5 and −0.5).

The following model was estimated for each outcome (i.e., child negative affect, parent negative affect, and parent–child relational strain):

The generic day-level (Level 1) equation was:

$$\begin{aligned} &{\mathrm{O}\mathrm{u}\mathrm{t}\mathrm{c}\mathrm{o}\mathrm{m}\mathrm{e}}_{ti}={\beta}_{00i}+ {\beta}_{01i}* {\mathrm{M}\mathrm{e}\mathrm{d}\mathrm{i}\mathrm{a} \left(\mathrm{w}\mathrm{i}\mathrm{t}\mathrm{h}\mathrm{i}\mathrm{n}\right)}_{ti}+\\&{\beta}_{02i}* {\mathrm{W}\mathrm{a}\mathrm{r} \left(\mathrm{w}\mathrm{i}\mathrm{t}\mathrm{h}\mathrm{i}\mathrm{n}\right)}_{ti}+{\beta}_{03i}* {\mathrm{C}\mathrm{h}\mathrm{i}\mathrm{l}\mathrm{d}\mathrm{c}\mathrm{a}\mathrm{r}\mathrm{e}\left(\mathrm{w}\mathrm{i}\mathrm{t}\mathrm{h}\mathrm{i}\mathrm{n}\right)}_{ti}+ {e}_{ti} \end{aligned}$$

Outcome(*ti*) is the predicted outcome (e.g., Parent negative affect) for family *i* on day *t*, which is predicted by this subject’s intercept (${\beta}_{00i}$), within-person variation in media use (${\beta}_{01i}$), War (within) (${\beta}_{02i}$), and by Childcare variable (yes/no) (${\beta}_{03i}$) plus a residual term quantifying the specific time-point deviation (${e}_{ti}$).

The generic person-level (Level 2) equations were:

$$\begin{aligned} &{\beta}_{0i}={\gamma}_{00}+{\gamma}_{01}*{\mathrm{M}\mathrm{e}\mathrm{d}\mathrm{i}\mathrm{a} \left(\mathrm{b}\mathrm{e}\mathrm{t}\mathrm{w}\mathrm{e}\mathrm{e}\mathrm{n} \right)}_{i}+ {\gamma}_{02}*{\mathrm{W}\mathrm{a}\mathrm{r} \left(\mathrm{b}\mathrm{e}\mathrm{t}\mathrm{w}\mathrm{e}\mathrm{e}\mathrm{n}\right)}_{i}+ \\& {\gamma}_{03}*{\mathrm{C}\mathrm{h}\mathrm{i}\mathrm{l}\mathrm{d} \mathrm{g}\mathrm{e}\mathrm{n}\mathrm{d}\mathrm{e}\mathrm{r}}_{i}+ {\gamma}_{04}*{\mathrm{P}\mathrm{a}\mathrm{r}\mathrm{e}\mathrm{n}\mathrm{t} \mathrm{g}\mathrm{e}\mathrm{n}\mathrm{d}\mathrm{e}\mathrm{r}}_{i}+ {\gamma}_{05}*{\mathrm{C}\mathrm{h}\mathrm{i}\mathrm{l}\mathrm{d} \mathrm{a}\mathrm{g}\mathrm{e}}_{i}+\\& {\gamma}_{06}*{\mathrm{P}\mathrm{a}\mathrm{r}\mathrm{e}\mathrm{n}\mathrm{t} \mathrm{i}\mathrm{n} \mathrm{m}\mathrm{i}\mathrm{l}\mathrm{i}\mathrm{t}\mathrm{a}\mathrm{r}\mathrm{y} \mathrm{s}\mathrm{e}\mathrm{r}\mathrm{v}\mathrm{i}\mathrm{c}\mathrm{e}}_{i}+ {u}_{0i}\end{aligned}$$

$${\beta}_{01i}={\gamma}_{010}+{u}_{01i}$$

$${\beta}_{02i}={\gamma}_{020}+{u}_{02i}$$

$${\beta}_{03i}={\gamma}_{030}+{u}_{03i}$$

In these equations, the intercept and the daily effects of media, and daily covariates were modeled using both fixed (γ00, γ10, γ20) and random parameters (µ1i, µ1i, µ2i) to account for between-family variability. In addition, the between-person effects of the media, exposure to war and the between-person covariates (Child and parent gender, child age, parent in military service) were included (γ01, γ02, γ03, γ04, γ05, γ06) as predictors in the equation of the intercept.

#### Mediation of The Relationship Between Parent Media Engagement and Child Negative Affect

If significant within/between-family associations were observed between parent media engagement and child negative affect, we tested whether these associations were mediated by parent negative affect or parent–child strain (i.e., Hypothesis 2). We utilized a multilevel structural equation model, employing the ‘lavaan’ package. This approach offers a robust analytic framework to estimate the mediation effects and allows for the estimation of relationships among variables within individuals and the variability in the strength of this effect across individuals. By incorporating random effects, it accounts for heterogeneity in the mediation pathway at the individual level.

#### Moderation of The Relationship Between Media Exposure and Outcomes

To examine the hypotheses concerning moderation effects (i.e., Hypotheses 3 and 5), we conducted an additional set of multilevel models. These models included direct exposure to war as a moderator, interacting with both within-person and between-person media variables. These interaction terms were incorporated at Level 2. Significant interaction effects were further scrutinized through analysis of simple slopes.

## Results

### Descriptive statistics

We first assessed the proportion of variance attributed to the two levels of analysis (Khan, 2021; Shi et al., 2021). Utilizing the intraclass-correlation coefficient (ICC), we quantified the extent of variance for variables assessed daily. Our analysis showed significant day-level fluctuations in the study variables. Specifically, for parental media engagement, the ICC was 54.6%, while for child media exposure, it was 50%. Daily objective exposure to war exhibited an ICC of 39.5%, while childcare showed 0% variability at the between-person level, indicating variability primarily within individuals across different days. Consequently, we determined that employing a multilevel modeling (MLM) technique was appropriate for testing the hypotheses (Chen et al., 2018). In addition, following Murayama et al. (2022), we also conducted a sensitivity analysis to evaluate statistical power given our sample size of 193 participants. The analysis indicated that the study had 80% power (α =.05) to detect small within-person effects (r >.10), as well as small between-person effects and cross-level interactions (r >.20).

Table 2 presents unweighted means, standard deviations, and bivariate correlations for the variables in the study, delineating between person-centered (within-person) and between-person mean correlations.

**Table 2:** Means, standard deviations, and within- and between-person correlations among the study variables

Variable	1	2	3	4	5	6	7
1. Parental negative affect	—	.20** [.14,.25]	.26** [.20,.32]	.19** [.13,.25]	.36** [.31,.41]		
2. Child negative affect	.58** [.48,.67]	—	.40** [.35,.45]	.08* [.02,.14]	.11** [.05,.16]		
3. Parent–child relational strain	.41** [.28,.52]	.66** [.57,.73]	—	.14** [.08,.20]	.21** [.15,.26]		
4. Media (child)	.19** [.05,.33]	.29** [.16,.42]	.19** [.05,.32]	—	.54** [.50,.58]		
5. Media (parent)	.14* [.00,.28]	.19** [.05,.32]	.18* [.04,.31]	.62** [.53,.70]	—		
6. Child age (months)	-.15* [-.28, -.01]	-.26** [-.39, -.13]	-.22** [-.35, -.09]	-.04 [-.18,.10]	-.06 [-.20,.08]	—	
7. Direct exposure to war	.28** [.14,.40]	.30** [.17,.42]	.07 [-.07,.21]	.22** [.08,.35]	-.04 [-.18,.11]	.28** [.15,.41]	—
*M*	12.44	7.32	7.17	6.78	10.22	53.45	4.63
*SD*	4.98	2.17	3.04	2.54	3.78	9.63	1.98
*Range*	6–30	5–25	3–20	5–34	5–49	36–71	0–12

*Notes. M* and *SD* are used to represent mean and standard deviation, respectively. Values in square brackets indicate the 95% confidence interval for each correlation. The confidence interval is a plausible range of population correlations that could have caused the sample correlation (Cumming, 2014). * indicates *p* <.05. ** indicates *p* <.01. The correlations between the person-centered (within-person) variables appear above the diagonal, and the correlations between-person mean (between-person) variables appear below the diagonal.

### The Impact of Parental Media Engagement on Parents and Children

Table 3 presents the findings from the multilevel models, with parental media engagement as the predictor variable and child negative affect, parent negative affect, and parent–child relational strain as the outcomes. Significant associations were observed between higher daily levels of parental media exposure (within-person effects) and all three outcomes. Specifically, increased parental media engagement was positively associated with elevated parent and child negative affect, and parent–child relational strain. Additionally, exposure to war emerges as a significant predictor of these outcomes. Notably, our analysis did not find significant between-person effects of parental media exposure.

**Table 3:** Multilevel model fixed effects for parental media usage predicting parent negative affect, child negative affect and parent–child relational strain

	**Parent negative affect**			**Child negative affect**			**Parent–child relational strain**		
*Predictors*	*Estimates*	*std. Error*	*p*	*Estimates*	*std. Error*	*p*	*Estimates*	*std. Error*	*p*
(Intercept)	14.41	0.65	** < 0.001**	8.15	0.33	** < 0.001**	7.12	0.41	** < 0.001**
Child gender [Girl]	−0.91	0.64	0.156	−0.20	0.28	0.480	0.05	0.40	0.895
Parent gender [Female]	2.09	0.68	**0.002**	0.17	0.30	0.558	0.73	0.43	0.091
Child age	−0.06	0.03	0.085	−0.06	0.01	** < 0.001**	−0.06	0.02	**0.003**
Direct exposure to war	0.46	0.17	**0.008**	0.26	0.07	**0.001**	0.36	0.11	**0.001**
Parent in military service [Yes]	1.88	1.17	0.112	1.22	0.51	**0.018**	−0.24	0.74	0.748
Daily direct exposure to war [Yes]	3.70	0.67	** < 0.001**	0.69	0.42	0.105	0.19	0.41	0.638
Childcare [No]	−1.82	0.31	** < 0.001**	0.06	0.17	0.726	−0.13	0.24	0.576
Media P Within	0.42	0.05	** < 0.001**	0.11	0.03	** < 0.001**	0.19	0.04	** < 0.001**
Media P Between	0.10	0.09	0.283	0.03	0.04	0.447	0.10	0.06	0.077
Conditional R^2^	0.727			0.559			0.575		
Marginal R^2^	0.215			0.123			0.113		

*Notes.* Conditional R^2^ and Marginal R.^2^ were computed based on Nakagawa et al.’s (2017) method using the*’performance’* package (Lüdecke et al., 2021)

We conducted a mediation analysis to examine whether parental media engagement is related to child negative affect through within-person effects. The results are summarized in Table 4.

**Table 4:** Multilevel mediation models examining parental negative affect and parent–child relational strain as mediators of the association between parental media engagement and child negative affect

	Mediator 1- Parental negative affect			Mediator 2- Parent–child relational strain		
*Fixed effects*	*Estimates*	*std. Error*	*p*	*Estimates*	*std. Error*	*p*
a	0.27	0.03	** < 0.001**	0.19	0.03	** < 0.001**
b	0.17	0.04	** < 0.001**	0.38	0.03	** < 0.001**
c (Direct effect)	0.10	0.03	0.071	0.03	0.03	0.285
Indirect effect	0.04	0.01	** < 0.001**	0.07	0.01	** < 0.001**
Total effect	0.10	0.03	** < 0.001**	0.10	0.03	** < 0.001**

*Notes*. a = effect of parental media engagement on mediator; b = effect of mediator on child negative affect; c = effect of parental media engagement on child negative affect. Indirect effect = a*b. Total effect = direct + indirect effect

**Fig. 1:**
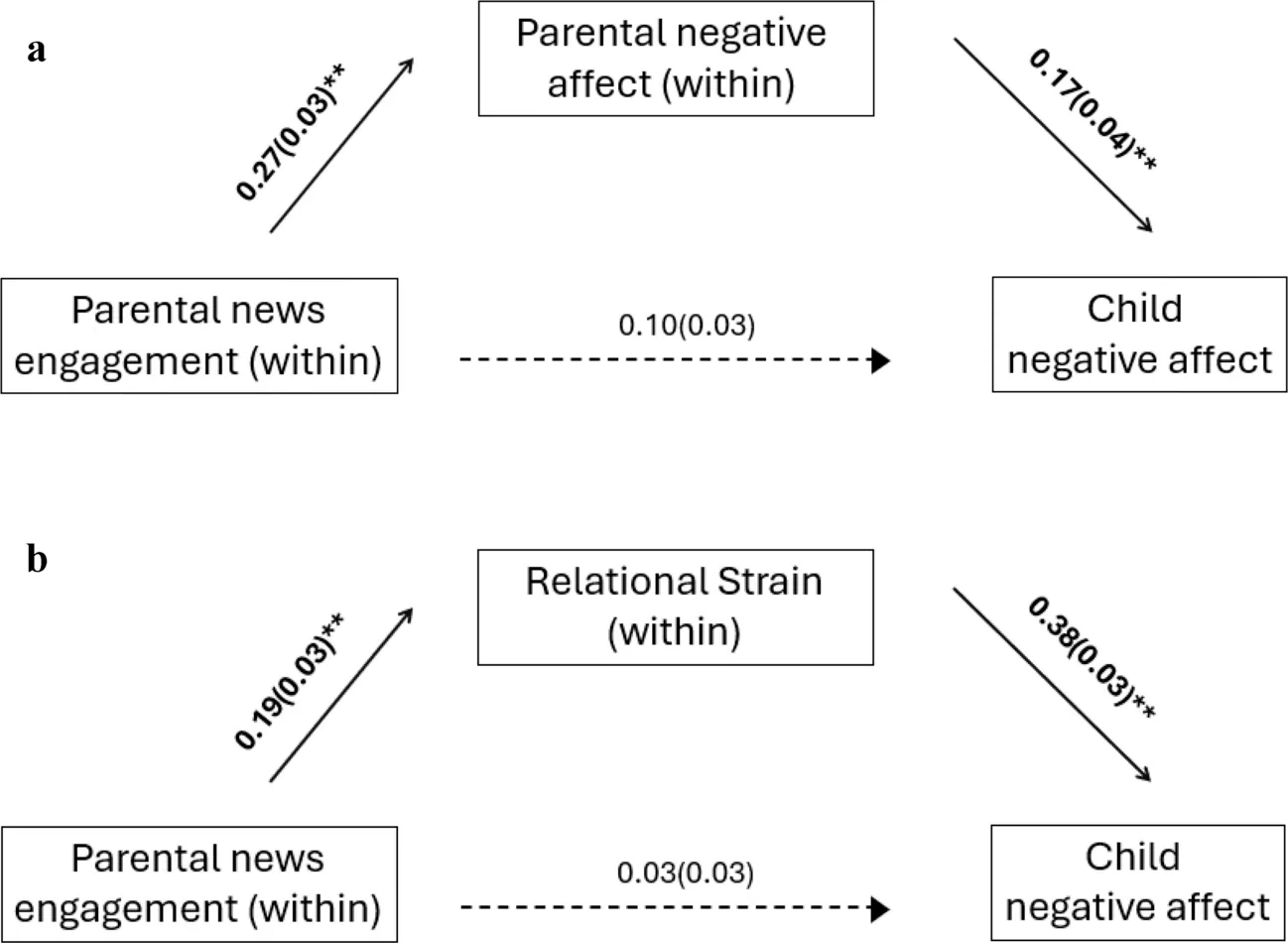
**a.** Mediation between parental news engagement and child negative affect by parental negative affect. **b.** Mediation between parental news engagement and child negative affect by parent–child relational strain

Our findings revealed significant within-person mediated effects, indicating that both parental negative affect and parent–child relational strain play intermediary roles in the relationship between parental media engagement and child negative affect. Notably, when including these mediators in the model, the direct effect of parental media engagement on child negative affect became non-significant, suggesting that the impact of parental media engagement on child negative affect is largely mediated by parental negative affect and parent–child relational strain. These results indicate that increased parental media engagement is associated with subsequent rises in parental negative affect and parent–child relational strain, leading to an increase in child negative behaviors (see Fig. 1.)

#### Moderation Effect of Exposure to War

The interactions between direct exposure to war and parental media engagement, both within-person and between-person effects, were not significant for the three outcomes (parental negative affect, child negative affect, and parent–child relational strain). This suggests that exposure to war does not moderate the effects of parental media engagement (see supplementary material).

### The Impact of Child Media Exposure on Children

Higher daily levels of child media exposure (within-person effects), higher average levels of media exposure (between-person effects), and exposure to war, were significant predictors of child negative affect and parent–child relational strain (see Table 5).

**Table 5:** Multilevel model fixed effects for child media exposure predicting child negative affect and parent–child relational strain

	Child negative affect			Parent–child relational strain		
*Predictors*	*Estimates*	*std. Error*	*p*	*Estimates*	*std. Error*	*p*
(Intercept)	8.03	0.32	** < 0.001**	7.13	0.41	** < 0.001**
Child gender [girls]	−0.19	0.27	0.494	0.11	0.40	0.787
Parent gender [Woman]	0.19	0.29	0.502	0.69	0.42	0.106
Child age	−0.06	0.01	** < 0.001**	−0.06	0.02	**0.004**
Direct exposure to War	0.27	0.07	** < 0.001**	0.39	0.11	** < 0.001**
Parent in military service [Yes]	1.06	0.49	**0.031**	−0.03	0.73	0.964
Direct exposure to War- day [Yes]	0.67	0.42	0.120	0.20	0.43	0.636
Childcare [No]	−0.12	0.16	0.454	−0.45	0.23	**0.046**
Media C Within	0.15	0.05	**0.003**	0.27	0.07	** < 0.001**
Media C Between	0.20	0.05	** < 0.001**	0.18	0.08	**0.025**
Conditional R^2^	0.540			0.571		
Marginal R^2^	0.152			0.114		

*Notes.* Conditional R^2^ and Marginal R.^2^ were computed based on Nakagawa et al. (2017) method using the*’performance’* package (Lüdecke et al., 2021)

#### Moderation Effect of Exposure to War

**Fig. 2:**
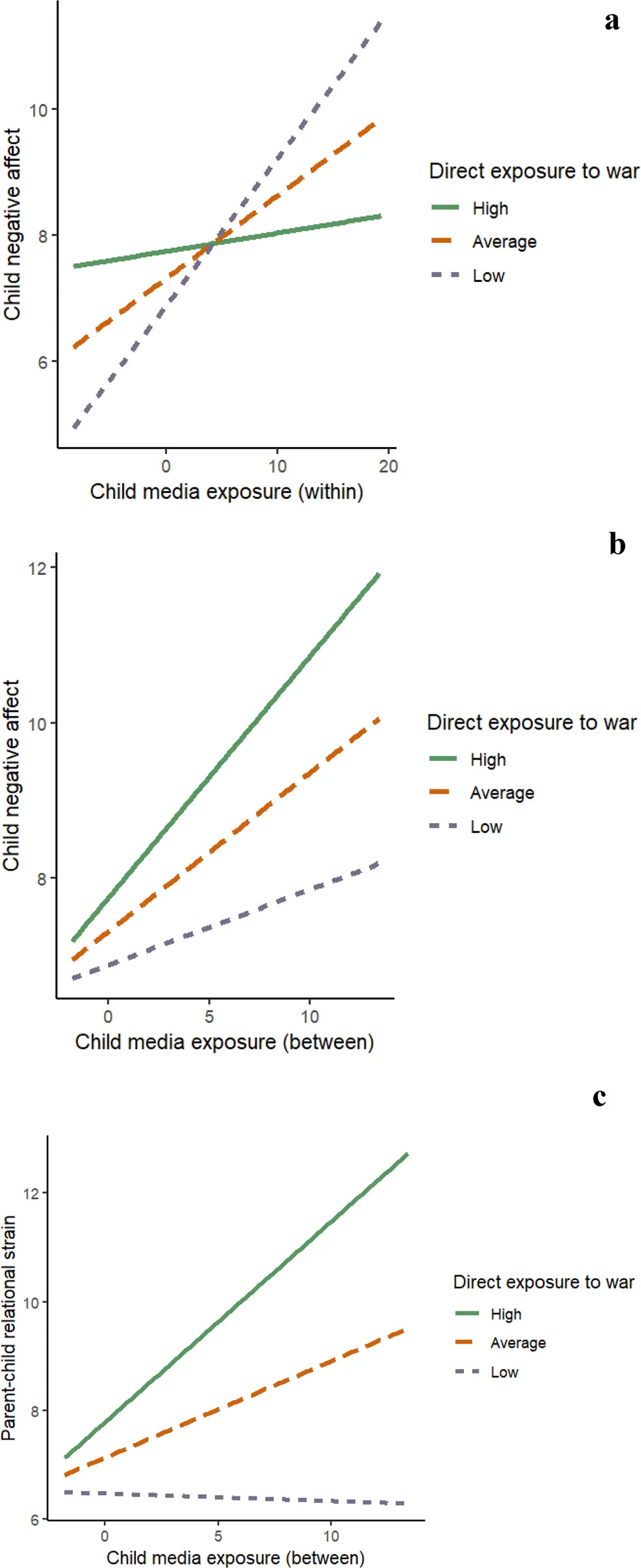
**a** The interaction between child war-related media exposure (within) and exposure to war in prediction of child negative affect. **b.** The interaction between child war-related media exposure (between) and direct exposure to war in prediction of child negative affect. **c.** The interaction between child war-related media exposure (between) and direct exposure to war in prediction of parent–child relational strain

Significant moderation effects were observed for direct exposure to war in the relationship between media exposure, both within-person and between-person, and child negative affect (see Table 6). Additionally, a significant moderation effect was found for direct exposure to war in the relation between between-person media exposure and parent–child relational strain (see Table 6). Analysis of simple slopes revealed that the relationship between daily media exposure and child negative affect was significant for individuals with low levels of war-exposure (Est. = 0.233, SE = 0.062, *p* =.0003) and average levels (Est. = 0.131, SE = 0.045, *p* =.0048), but not for those with high levels of exposure (Est. = 0.029, SE = 0.055, *p* =.603) (see Fig. 2a). Conversely, the relationships between media exposure and child negative affect and parent–child relational strain were significant for individuals with high (respectively, Est. = 0.311, SE = 0.074, *p* <.0001; Est. = 0.368, SE = 0.109, *p* =.001), and average levels of war exposure (respectively, Est. = 0.204, SE = 0.054, *p* =.0002; Est. = 0.177, SE = 0.081, *p* =.0292), but not for those with low (respectively, Est. = 0.097, SE = 0.074, *p* =.188; Est. = −0.013, SE = 0.111, *p* =.901) levels of exposure (refer to Fig. 2b and 2c).

**Table 6:** Multilevel model fixed effects for the interaction between child’s media exposure and war

Child negative affect				Parent–child relational strain		
*Predictors*	*Estimates*	*std. Error*	*p*	*Estimates*	*std. Error*	*p*
(Intercept)	7.91	0.32	** < 0.001**	6.97	0.41	** < 0.001**
Parent gender [Woman]	−0.25	0.28	0.375	−0.78	0.42	0.067
Child gender [Girl]	0.15	0.27	0.566	−0.17	0.40	0.672
Child age	−0.06	0.01	** < 0.001**	−0.07	0.02	**0.001**
Parent in military service [Yes]	−0.84	0.49	0.088	0.33	0.73	0.649
Daily direct exposure to war [Yes]	−0.66	0.42	0.118	−0.21	0.43	0.618
Childcare [No]	−0.12	0.16	0.449	0.47	−0.22	**0.038**
Direct exposure to war	0.22	0.07	**0.003**	0.33	0.11	**0.002**
Media C Within	0.13	0.04	**0.004**	0.26	0.07	** < 0.001**
Media C Between	0.20	0.05	** < 0.001**	0.18	0.08	**0.026**
Media C Within × Direct exposure to War	−0.05	0.02	**0.008**	0.03	0.03	0.372
Media C Between × Direct exposure to War	0.05	0.02	**0.030**	0.10	0.04	**0.011**
Conditional R^2^	0.527			0.573		
Marginal R^2^	0.161			0.130		

*Notes.* Conditional R^2^ and Marginal R.^2^ were computed based on Nakagawa et al.’s (2017) method using the*’performance’* package (Lüdecke et al., 2021)

## Discussion

This study examined daily associations between war-related media exposure on children and parents during ongoing regional conflict. We explored how direct and indirect war exposure, and their interaction, relate to parent and child negative affect and parent–child relational strain, with a particular focus on fluctuations in daily news media exposure. Our study revealed significant associations between parental and child news media exposure, and parental reports of child negative affect, parental negative affect, and parent–child relational strain. Specifically, our findings showed that parental news media engagement had a notable within-subject impact on both parental and child emotional state, while child media exposure emerged as a significant factor affecting emotional state with both within and between effects. Moreover, parental media engagement was associated with child outcomes through two mediating pathways: parental negative affect and parent–child relational strain, within subjects. We also found interactions between direct and indirect exposure to war for children, but not for parents, indicating different meanings for between-subjects and within-subjects analyses. These findings illuminate the intricate relationships between media exposure, war-related events exposure, and parent–child emotional and interactional processes in the current era, providing insights into potential interventions and policy development to support families during regional conflict.

### The Impact of Parental Media Engagement on Parent and Child

First, our analysis revealed that higher daily levels of parental news media engagement (within-person effects) were significantly associated with increased parental and child negative affect and parent–child relational strain. Notably, direct exposure to war was also a significant predictor of these outcomes. While our findings align with previous research demonstrating the detrimental effects of direct and indirect exposure to terrorism on mental well-being (Monfort & Afzali, 2017; Silver et al., 2013; Slone & Shoshani, 2022), we specifically examined daily negative affect, which provides insight into the immediate emotional responses of parents and children. Future studies should examine the long-term implications of exposure to war and address wider aspects of emotional well-being and mental health outcomes.

Moreover, our study found that parental media news engagement not only impacts parents’ negative affect but also influences children’s negative affect and the dynamics of child-parent interaction. This suggests that parental media engagement shapes the emotional climate within the parent–child relationship. Specifically, higher daily levels of parental media engagement may contribute to heightened negative emotions and strained interactions between parents and children on a daily basis. These insights underscore the importance of considering the broader parent–child relational processes when examining the effects of media exposure (Coyne et al., 2014), especially during times of conflict and war, where both media exposure and parent–child interaction play crucial roles in shaping children’s emotional responses and overall well-being. Previous studies have suggested that exposure to television news can evoke fear and anxiety reactions in children (Cantor, 2001; Riddle et al., 2012). Our findings extend this perspective by showing that even indirect exposure, through parents’ news consumption, may have a cascading effect, leading to sadness, irritability, tearfulness, tantrums, and anxiety in children. This underscores the importance of supporting families facing these challenges with tailored interventions, especially during times of conflict (Slone et al., 2008).

Secondly, our study delved into the mechanisms underlying the significant within-person relationship between parents’ media engagement and child negative affect. We uncovered within-person mediating effects, indicating that both parental negative affect and parent–child relational strain play distinct mediating roles.

These findings provide novel insights into the intricate relationship between parental media news engagement and child well-being. To the best of our knowledge, our research represents one of the initial attempts to explore how parental media exposure to news influences their children emotional state, shedding light on previously unexplored pathways through which parental media engagement may impact child outcomes. This suggests that parental media engagement impacts children’s emotional responses through multiple pathways, including parent–child interaction processes and direct emotional transmission. Future studies should deeper explore the differences in the impacts of these mechanisms. Furthermore, these findings not only enhance our understanding of dynamics within conflict-affected families but also offer valuable insights for intervention and support strategies.

### The Impact of Child Media Exposure on the Child

Elevated daily levels of children’s news media exposure (within-person effects), higher average levels of news media exposure (between-person effects), and direct exposure to war, emerged as predictors of children’s negative affect and parent–child relational strain. These findings align with previous research on the impact of disaster and terrorism exposure via television (Otto et al., 2007; Comer et al., 2007). However, prior studies primarily focused on older age groups and often one-off events. In contrast, our study highlights the effects of media exposure during an ongoing conflict, particularly among younger children, emphasizing its influence on daily emotional states and overall well-being. Our findings expand Cantor and Wilson theories (1984), which suggest that older children may be particularly susceptible to violent content, and underscore the strong impact of media exposure on preschoolers as well particularly in the context of war-related media. Furthermore, the findings emphasize the importance of caregivers’ attention to children’s exposure to war content, even indirectly. Despite these insights, further research is needed to explore how children process and are affected by news media exposure, with possible moderators (e.g., parental mediation).

Importantly, within-person and between-person effects reflect different aspects of media exposure during ongoing conflict. Between-person effects capture more stable differences in individuals’ overall levels of media engagement, whereas within-person effects capture day-to-day fluctuations in media exposure and their short-term associations with emotional responses. When interpreting the findings, it’s essential to acknowledge the nuanced differences in media exposure effects between parents and children. Interestingly, our study revealed that while media exposure had a within-subjects effect for parents, it had both within-subjects and between-subjects effects for children. This pattern suggests that children may be influenced not only by day-to-day increases in media exposure but also by more stable differences in their overall level of exposure. This distinction can be attributed to several factors. Firstly, adults possess more robust regulatory mechanisms (Sabatier et al., 2017), allowing them to return to a state of normalcy after indirect exposure to terrorism. In contrast, children, with their still-developing emotional regulation skills, may struggle to process and regulate such exposure, resulting in longer-lasting effects on their well-being. Indeed, research indicates that children can experience prolonged distress in response to fear-inducing media, as they often have difficulty distinguishing between real and imagined threats, leading to heightened fear responses that can persist over time (Cantor, 2001). Additionally, individual differences play a significant role in how people use media for emotional regulation, particularly during periods of negative affect like fear or stress (Schramm & Cohen, 2017). These coping styles influence media selection for emotion regulation. Children, who do not choose to be exposed to news media themselves and are exposed to threats during ongoing conflict, may be even more influenced by the media. Another plausible explanation could be that a between-subjects effect exists for adults as well, albeit weaker. With a larger sample size and greater statistical power, such an effect may have been detectable among adults.

Moreover, the within-subject effect of indirect exposure to terrorism through the media, an aspect that has received limited attention in prior research, underscores the profound implications of reducing media consumption, even intermittently. This highlights the importance of considering not just the overall volume of exposure but also the fluctuations in exposure over time, particularly in the context of ongoing conflict and heightened media coverage.

### Interplay Between War Exposure and Media Exposure

Our study highlights the interplay between direct and indirect exposure to war and their impact on both children and adults. Significantly, we observed a significant interaction effect between direct exposure to war and media exposure, which was evident only in children. Children, unlike adults, have a developing capacity to differentiate between real and perceived threats, which may amplify their distress when exposed to intense media coverage of conflicts (Wilson, 2008). This age group’s developing cognitive and emotional resources leave them particularly susceptible to distressing newsThis vulnerability highlights the need for targeted interventions to mitigate the psychological impact of both direct and indirect exposures on preschoolers during times of conflict.

Moreover, our findings highlighted that the significant moderating effects of war exposure on the relationship between media exposure and child negative affect. Similarly, war exposure moderated the association between news exposure and parent–child relational strain, only between subjects. Consistent with our hypotheses, children’s elevated direct exposure to war amplified the negative effects of media exposure. Conversely, within-subject analyses revealed a different pattern, suggesting that children with lower direct exposure were more susceptible to fluctuations in media exposure. These findings emphasize the importance of considering media exposure in the context of ongoing conflict. While cumulative media exposure exacerbates the negative effects of direct exposure among highly exposed children, daily fluctuations in media usage have a stronger influence on individuals with lower direct exposure. These insights underscore the multifaceted nature of media exposure during conflict and the need to address the differential vulnerabilities of children based on their levels of direct war exposure.

### Limitations and Conclusions

While our study presents valuable insights, it’s important to acknowledge several limitations. Firstly, our study relied on self-report measures, which are susceptible to biases such as social desirability and memory recall. Incorporating objective measures in future studies could complement the self-report data. Additionally, our sample size should be noted as a potential limitation, as diary studies typically require larger samples to achieve adequate statistical power. In addition, because the analyses examined same-day associations between media exposure and emotional outcomes, the temporal ordering between these variables cannot be established. Therefore, the findings should be interpreted as associations rather than causal effects. Future research using lagged designs could better clarify the directionality of these relationships. The present study also focused on the parent–child dyad and did not capture broader family-level processes involving multiple family members. Future research should examine how war-related media exposure operates within the broader family system.

Moreover, our sample only included participants that identified as Jewish-Israeli, and therefore our findings cannot be generalized on the general population in Israel, that includes a 21% Arab-Muslim minority. This homogeneity in the sample limits the ability to understand the full range of experiences, particularly in light of the ethnic and sociopolitical diversity in the country. While we recognize that our study did not capture the perspectives of non-Jewish populations, which may have experienced the conflict differently, we believe that understanding the experiences of the Jewish-Israeli population during the conflict provides a first step toward contextualizing the broader implications of media exposure in such contexts.

Finally, while our study focused on the impact of media exposure on parent and child well-being during conflict, it did not explore potential moderating factors such as parental mediation strategies (Swider-Cios et al., 2023) or children’s coping mechanisms (Varela et al., 2022). In addition, the measure of children’s exposure captured the time children were present with parents during news consumption but did not distinguish between different forms of exposure (e.g., direct visual exposure, auditory exposure, or being present in the same space without actively attending to the content). Notably, disentangling these forms of exposure is inherently challenging given the dynamic and unpredictable nature of young children’s behavior — young children move frequently between spaces, shift attention rapidly, and may engage with or disengage from media content in ways that are difficult for parents to monitor and retrospectively report with precision. Future research could move beyond parental self-report by employing observational methods, experience sampling, or device-based tracking to more accurately capture the nature and quality of young children’s exposure to war-related media in family contexts. Exploring these factors in future research could provide a more comprehensive understanding of the mechanisms underlying the relationship between media exposure and child outcomes. Additionally, the measure of parent–child relational strain used in the current study may not fully capture broader parenting and parent–child interaction processes. Future research should employ more comprehensive measures of parenting, parental stress, and parent–child interactions to further clarify these mechanisms.

Beyond these limitations, another factor that may shape responses to war-related media exposure is individuals’ pre-existing mental health symptoms. Future research should examine whether prior emotional vulnerabilities among parents or children may moderate the associations between war-related media engagement and daily emotional responses. Examining such moderating factors may also have important implications for intervention efforts. Identifying families who may be particularly vulnerable to the emotional impact of war-related media exposure could help inform targeted support strategies at the individual and family levels, such as parent guidance on monitoring and regulating children’s exposure to distressing news content and increasing awareness of how parents’ own media engagement may shape children’s emotional responses. In addition, future research may inform broader structural or policy-level recommendations regarding media consumption during periods of ongoing conflict.

The present study contributes to the literature by examining the interplay between news media exposure, war exposure, and parent–child relational processes during ongoing conflict, specifically among young children. The findings highlight the role of parental media engagement in shaping children’s emotional experiences and underscore the importance of considering both direct and indirect exposure to war-related information in family contexts. Furthermore, our study sheds light on the nuanced interaction between direct and indirect exposure to war for children, suggesting that day-to-day fluctuations in media exposure and more stable differences in overall exposure may play different roles in children’s emotional responses.

## Supplementary Information

Below is the link to the electronic supplementary material.

Supplementary file1 (DOCX 36 KB)

## Data Availability

The datasets generated and analyzed during the current study are not publicly available due to ethical considerations but are available from the corresponding author upon reasonable request.
