# Supplementary material for: Media News Engagement and Parent–Child Well-being during Regional Conflict: A Daily Diary Study
Source: Res Child Adolesc Psychopathol. 2026 Jun 17;54(4):77. doi: 10.1007/s10802-026-01470-x (PMC13275578; doi:10.1007/s10802-026-01470-x)
Supplement: Supplementary file 1 — Supplementary file1 (DOCX 36 KB) [file 10802_2026_1470_MOESM1_ESM.docx]

**Supplementary Material**

**Exposure to War Questionnaire**

|  | **Happened to me** | **Did not happen to me** |
| --- | --- | --- |
| We had to cancel a certain activity due to security threats. |  |  |
| My child did not attend their educational setting due to security threats. |  |  |
| There was rocket fire and a rocket alarm sounded while my child was in their educational setting. |  |  |
| Rocket alarms were activated in our residential area. |  |  |
| A rocket or rocket fragment fell nearby, but no one was injured. |  |  |
| A household member is serving in reserve duty or in the regular army in a combat role. |  |  |
| My family had to leave our home due to security threats. |  |  |
| Terrorists infiltrated our residential area. |  |  |
| Our home was damaged by a rocket or gunfire. |  |  |
| My child was directly exposed to a terror event. |  |  |
| I or a household member was directly exposed to a terror event. |  |  |
| My child was injured in the current war. |  |  |
| I or someone close to me was injured in the current war. |  |  |
| Someone close to me was abducted in the current war. |  |  |
| Someone I know (not close to me) was abducted in the current war. |  |  |
| Someone close to me was killed in the current war. |  |  |
| Someone I know (not close to me) was killed in the current war. |  |  |
| Has your family experienced a war-related event not listed here? If so, please specify _____ |  |  |

Below is a list of events related to the security situation. For each event, please circle whether it has happened to you or your family since the beginning of the war.

**Table 1 - Stepwise Regression Models**

Stepwise regression for the selection of covariates to include in each model

| **Predictor** | **Model 1**  for ‘Child Negative Affect’ | | | **Model 2**  for ‘Parent Negative Affect’ | | | **Model 3**  for ‘Conflictual Interaction’ | | |
| --- | --- | --- | --- | --- | --- | --- | --- | --- | --- |
|  | **In final model** | **Coefficient** | ***p*** | **In final model** | **Coefficient** | ***p*** | **In final model** | **Coefficient** | ***p*** |
| *Minimal model* | *Variables retained in the final model based on theoretical considerations* | | | | | | | | |
| **(Intercept)** | **(✓)** | **9.8111** | **< 2e-16 ***** | **(✓)** | **7.845** | **< 8-014***** | **(✓)** | **11.538** | **< 2e-16 ***** |
| **Parent sex[woman]** | (✓) | 0.427 | 0.164 | **(✓)** | **2.76** | **0.000 ***** | **(✓)** | **1.009** | **0.015*** |
| **Child sex [girl]** | (✓) | -0.085 | 0.772 | (✓) | -1.918 | 0.056 . | (✓) | 0.005 | 0.988 |
| *Considered predictors* | *Variables considered in stepwise regression* | | | | | | | | |
| **Child age** | **✓** | **-0.052** | **0.000 ***** | ✗ | --- | --- | **✓** | **-0.0622** | **0.004 **** |
| Parent education | ✗ | --- | --- | ✗ | --- | --- | ✗ | --- | --- |
| Parent age | ✗ | --- | --- | ✗ | --- | --- | ✗ | --- | --- |
| Parent occupation | ✗ | --- | --- | ✗ | --- | --- | ✗ | --- | --- |
| Categorical geographic location | ✗ | --- | --- | ✗ | --- | --- | ✗ | --- | --- |
| Family marital status | ✗ | --- | --- | ✗ | --- | --- | ✗ | --- | --- |
| Salary | ✗ | --- | --- | ✗ | --- | --- | ✗ | --- | --- |
| Educational setting of child (type) | ✗ | --- | --- | ✗ | --- | --- | ✗ | --- | --- |
| Number of children in the family | ✗ | --- | --- | ✗ | --- | --- | **✓** | **-0.513** | **0.002 **** |
| Birth order | ✗ | --- | --- | ✗ | --- | --- | ✗ | --- | --- |
| **Parent in military service** | **✓** | **1.429** | **0.007 *** | **✓** | **---** | **---** | ✗ | --- | --- |
| **Childcare [day- no]** | ✗ | **---** | **---** | **✓** | **1.857** | **0.040*** | **✓** | **-0.561** | **0.015*** |
| Childcare (hours) | ✗ | **---** | **---** | **✓** | **0.649** | **2e-07 ***** | ✗ | --- | --- |

**Table 2**

Multilevel model fixed effects for the interaction between parental news media engagement and war.

|  | **Parent Negative Affect** | | | **Child Negative Affect** | | | **Conflictual Parent-Child Interaction** | | |
| --- | --- | --- | --- | --- | --- | --- | --- | --- | --- |
| *Predictors* | *Estimates* | *std. Error* | *p* | *Estimates* | *std. Error* | *p* | *Estimates* | *std. Error* | *p* |
| (Intercept) | 14.36 | 0.67 | **<0.001** | 8.05 | 0.34 | **<0.001** | 7.03 | 0.42 | **<0.001** |
| Child sex [Girl] | -2.12 | 0.69 | **0.002** | -0.26 | 0.30 | 0.383 | -0.80 | 0.43 | 0.067 |
| Parent sex[Woman] | 0.92 | 0.64 | 0.154 | 0.21 | 0.28 | 0.443 | -0.05 | 0.40 | 0.904 |
| Child age | -0.06 | 0.03 | 0.081 | -0.06 | 0.01 | **<0.001** | -0.07 | 0.02 | **0.002** |
| Direct exposure to War | 0.44 | 0.18 | **0.014** | 0.23 | 0.08 | **0.003** | 0.35 | 0.11 | **0.002** |
| Parent in military service [Yes] | -1.83 | 1.19 | 0.126 | -1.03 | 0.51 | **0.046** | 0.34 | 0.75 | 0.648 |
| War- day [Yes] | -3.69 | 0.67 | **<0.001** | -0.75 | 0.42 | 0.080 | -0.19 | 0.41 | 0.638 |
| Childcare [No] | 1.81 | 0.31 | **<0.001** | -0.07 | 0.17 | 0.684 | 0.14 | 0.24 | 0.552 |
| Media P Within | 0.43 | 0.05 | **<0.001** | 0.11 | 0.03 | **<0.001** | 0.19 | 0.04 | **<0.001** |
| Media P Between | 0.09 | 0.09 | 0.310 | 0.02 | 0.04 | 0.592 | 0.09 | 0.06 | 0.112 |
| Media P Within × Direct exposure to War | -0.01 | 0.02 | 0.584 | -0.01 | 0.01 | 0.313 | 0.01 | 0.02 | 0.707 |
| Media P Between × Direct exposure to War | 0.01 | 0.04 | 0.793 | 0.03 | 0.02 | 0.075 | 0.03 | 0.03 | 0.332 |
| Conditional R^2^ | 0.727 |  |  | 0.559 |  |  | 0.577 |  |  |
| Marginal R^2^ | 0.212 |  |  | 0.123 |  |  | 0.116 |  |  |

*Notes.* Conditional R^2^ and Marginal R.^2^ were computed based on Nakagawa et al. (2017) method using the *‘performance’* package (Lüdecke et al., 2021)
